# Prevalence and phylogenetic analysis of hemoplasma species in domestic pigs in Korea

**DOI:** 10.1186/s13071-019-3638-x

**Published:** 2019-07-29

**Authors:** Min-Goo Seo, Oh-Deog Kwon, Dongmi Kwak

**Affiliations:** 1 Animal and Plant Quarantine Agency, 177 Hyeoksin 8-ro, Gimcheon, Gyeongbuk 39660 South Korea; 2 College of Veterinary Medicine, Kyungpook National University, 80 Daehakro, Bukgu, Daegu, 41566 South Korea

**Keywords:** *Mycoplasma parvum*, *M*. *suis*, Novel hemotropic *M*. *haemosuis*, Phylogeny, Pig

## Abstract

**Background:**

Two hemoplasma species, *Mycoplasma suis* and *M. parvum*, previously known as *Eperythrozoon suis* and *E. parvum*, respectively, have been identified in pigs. Swine hemoplasmosis is a global problem, and *M. suis* infection results in economic losses to pig producers worldwide. This study investigated the frequency and distribution of hemotropic mycoplasmas in pig farms of Korea. As hemoplasmas can be transmitted by ticks, we also analyzed the presence of the tick-borne pathogens *Anaplasma* spp. and *Borrelia* spp.

**Methods:**

We screened 1867 samples from 464 pig farms located in four regions of Korea over the period from 2014 to 2018. PCR-positive samples were further analyzed by nucleotide sequencing and phylogenetic analysis of pathogen-specific markers for species identification.

**Results:**

Of the 1867 pigs evaluated in the study, three (0.2%), 51 (2.7%), and one (0.1%) were found to be infected with *M. suis*, *M. parvum*, and the novel hemotropic *M. haemosuis*, respectively; *Anaplasma* spp. and *Borrelia* spp. were not detected. The *16S* rRNA sequences of *M. suis*, *M. parvum*, and the novel hemotropic *M. haemosuis* were highly similar (99.3–100%, 99.6–100%, and 99.6–100%, respectively) to those of *Mycoplasma* spp. isolated from other countries. To the best of our knowledge, this is the first nationwide, large-scale study of the molecular detection of *Mycoplasma* spp. in domestic pigs in Korea.

**Conclusions:**

Our results indicate that *Mycoplasma* infections are widespread in Korean domestic pigs, and that continuous monitoring and control strategies are required to prevent the spread of hemoplasmas, which, in addition to causing economic losses in the pig industry, pose a potential threat to public health. As transmission routes of hemoplasmas remain unelucidated, additional epidemiological studies are recommended to identify reservoirs and vectors of *Mycoplasma* spp. in Korea.

## Background

Hemoplasma refers to a group of hemotropic prokaryotes that lack a cell wall and are uncultivable *in vitro* [1]. Two hemoplasma species, *Mycoplasma suis* and *M. parvum*, previously known as *Eperythrozoon suis* and *E. parvum*, respectively, have been previously identified in pigs [2]; these two organisms can be distinguished based on morphology, pathogenicity [3], and mode of interaction with the host.

*Mycoplasma suis*, a small, rickettsia-like, rod-shaped, extracellular pleomorphic microorganism, which attacks erythrocytes [4] and is the causative agent of hemoplasmosis (previously called eperythrozoonosis) in domestic pigs [5]. This species was previously known as *M. haemosuis* (basonym *E. suis*) or “*Candidatus* M. haemosuis”, indicating the provisional status of an incompletely described taxon [6, 7]. Clinical signs of *M. suis* infection in pigs vary, but acute infection manifests as icterus and febrile hemolytic anemia and is characterized by low morbidity but high mortality rates [1]. Chronic *M. suis* infection in pigs causes growth delay and has been associated with low reproductive efficiency and an increased incidence of other infectious diseases [1, 8]. Pigs infected with *M. suis* are likely to be persistent chronic carriers of the pathogen even after the resolution of clinical signs [9].

*Mycoplasma parvum* is the last validly published species in the genus *Eperythrozoon*, *E. parvum*. This species is not available and there is no assurance that it can be found in a reasonable amount of time [6]. Since all the other known *Eperythrozoon* species have now been shown to be *Mycoplasma* species, it seems likely that *E. parvum* would prove to belong to the genus *Mycoplasma*. However, there is no direct proof and it cannot be decided when a sample of this bacterium can be found and studied [6]. Clinical signs of *M. parvum* infection are less well-known, and it is currently believed that this organism exerts relatively low pathogenicity in pigs. It has been shown that *M. parvum* was not associated with clinical signs even at the peak of bacteremia in intact piglets, and that severe anemia with pyrexia was detected only in splenectomized piglets [3, 10, 11].

Swine hemoplasmosis is a global disease, and *M. suis* infection causes economic losses for pig producers worldwide [1]. However, despite its evident economic importance, *M. suis* in pig herds is rarely reported, and its infection is regularly under-diagnosed or not diagnosed, which can lead to significant production losses [9]. To date, no information is available on hemotropic mycoplasmas specific to Korean pigs. Several *Mycoplasma* spp. have been detected by PCR and reported in other countries in pigs, such as *M. suis*/*M. parvum* and, the novel hemotropic *M. haemosuis* in China [12], *M. suis* and *M. parvum* in Japan [13], *M. suis* in China [14], *M. suis* in wild boars [15] and pigs [9] in Germany, and *M. suis* in Brazil [16]. This study thus investigated the frequency and distribution of hemotropic mycoplasma species in domestic pig farms of Korea. As *M. suis* can be transmitted by ticks, we also analyzed the presence of the tick-borne pathogens *Anaplasma* spp. and *Borrelia* spp., which has not been performed before in domestic pigs.

## Methods

### Sample size determination and sample collection

The total number of pigs raised in 6196 farms in Korea in 2018 was recorded at 11,640,677 [17]. In this study, we used simple random sampling, and sample size was determined by power analysis using an expected disease prevalence of 10%, accepted absolute error of 5%, and confidence level of 99% [18], as follows:

$$ n\, = \,{{ 2. 5 8^{ 2} p_{ \exp } \left( { 1- p_{ \exp } } \right)} \mathord{\left/ {\vphantom {{ 2. 5 8^{ 2} p_{ \exp } \left( { 1- p_{ \exp } } \right)} {d^{ 2} }}} \right. \kern-0pt} {d^{ 2} }} $$

where *n* is the required sample size, *p*_exp_ is the expected prevalence, and *d* is the desired absolute precision.

According to the formula, a minimum of 239 samples was required. We randomly selected 1867 samples from 464 pig farms located in four regions of Korea. Blood was collected from the jugular vein, and breed and region were recorded.

### PCR

Genomic DNA was extracted from whole blood samples using the DNeasy Blood and Tissue Kit (Qiagen, Melbourne, Australia) according to the manufacturer’s protocol, and its quantity and quality were measured using a NanoDrop™ 2000 spectrophotometer (Thermo Fisher Scientific, Wilmington, DE, USA) before storage at − 20 °C until analysis.

Screening was performed by nested PCR using the AccuPower HotStart PCR Premix Kit (Bioneer, Daejeon, Korea) and designated primer sets. *Anaplasma* spp. were detected based on amplification of the *16S* rRNA gene using the primer sets EE1/EE2 and EE3/EE4 [19]. *Borrelia* spp. were identified based on the presence of the 5S (*rrf*)–23S (*rrl*) intergenic spacer using primer sets Bb23S3/Bb23Sa and Bb23SnF/Bb23SanR, and *B. burgdorferi* was detected by amplification of the outer surface protein A gene fragment using primer sets N1/C1c and N2/C2c [20]. Hemoplasmas were first identified based on the amplification of *16S* rRNA with universal primers fHf1/rHf2 and *M. suis*-specific primers f2/r2 [16, 21]; positive results were then confirmed at the species level by PCR using cmsf2/cmsr2 and msf2/msf2 primer sets to amplify the *16S* rRNA gene of *M. suis*, *M. parvum*, and the novel hemotropic *M. haemosuis* [12].

### DNA cloning

Amplified *16S* rRNA gene fragments were purified using the QIAquick Gel Extraction Kit (Qiagen) inserted into the pGEM-T Easy vector (Promega, Madison, WI, USA) following the manufacturer’s instructions, and the resulting constructs were used to transform *Escherichia coli* DH5α-competent cells (Thermo Fisher Scientific). Bacteria were incubated at 37 °C overnight, and plasmids were purified using a plasmid miniprep kit (Qiagen) according to the manufacturer’s instructions.

### DNA sequencing and phylogenetic analysis

Recombinant plasmids were sequenced by Macrogen (Seoul, Korea), and *16S* rRNA gene sequences were analyzed using the multiple sequence alignment program CLUSTAL Omega (ver. 1.2.1). Results of sequence alignment were corrected using BioEdit (ver. 7.2.5), and phylogenetic analysis was performed with MEGA (ver. 6.0) using the maximum likelihood method based on the Kimura 2-parameter distance model. The aligned sequences from the present study and those available from the GenBank database were analyzed using a similarity matrix. The stability of the obtained phylogenetic trees was estimated by bootstrap analysis with 1000 replicates.

### Statistical analysis

The analytical software package GraphPad Prism version 5.04 (GraphPad Software Inc., La Jolla, CA, USA) was used for statistical analysis. The chi-square test was applied to assess significant differences between groups, and a *P*-value of less than 0.05 was considered statistically significant. Confidence intervals (95% CI) were calculated for all estimates.

## Results

### PCR detection

**Fig. 1:**
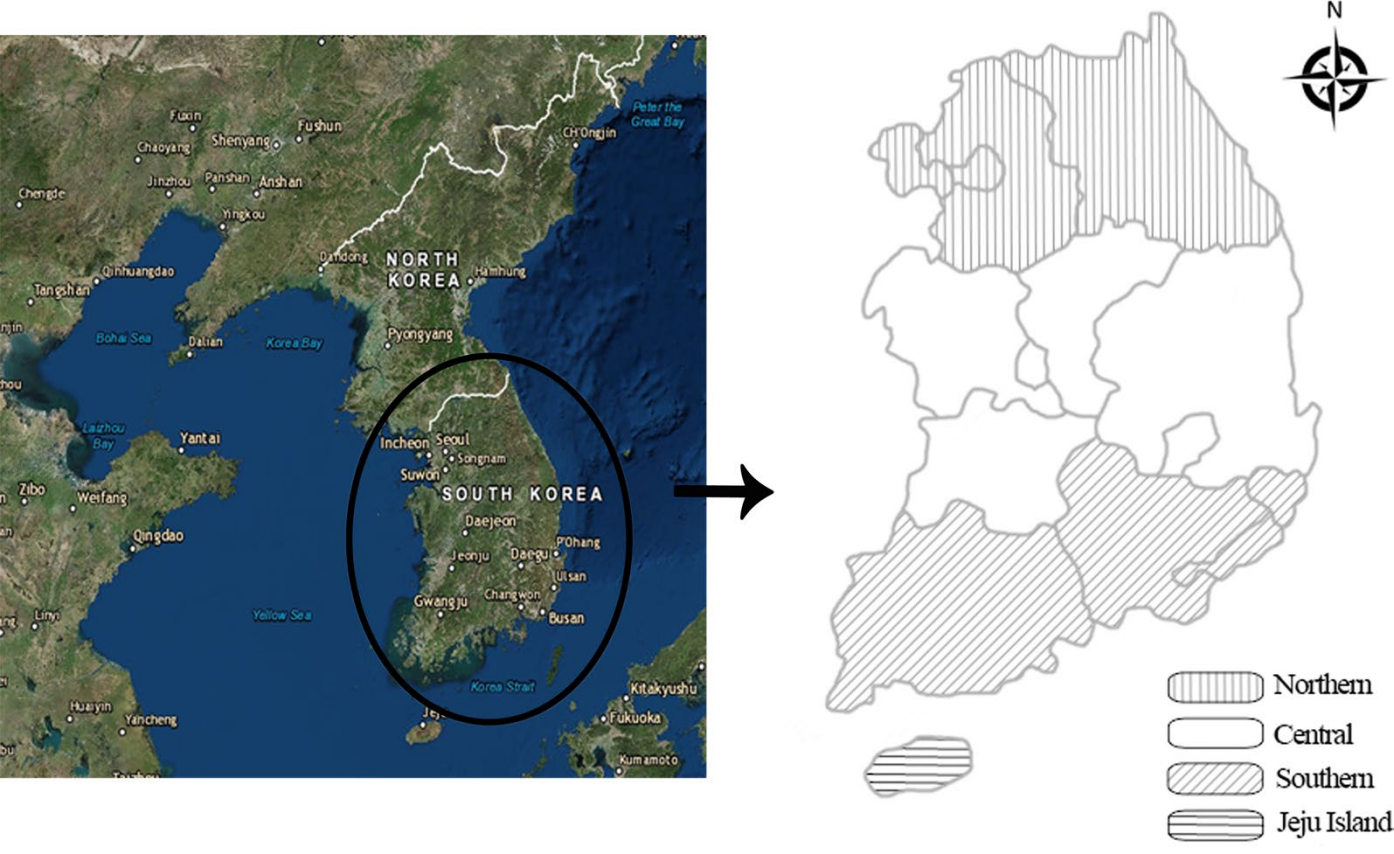
The four regions of Korea (Northern, Central, Southern and Jeju Island) where pig blood samples were collected to detect hemoplasmas

We randomly selected 1867 samples from 464 pig farms located in four regions of Korea (Fig. 1). PCR amplification of *16S* rRNA gene fragments showed that 55 of the 1867 pigs tested (2.9%) were positive for *Mycoplasma* spp. Additional PCR analysis conducted on the 55 positive samples to identify *M. suis*, *M. parvum* and the novel hemotropic *M. haemosuis* species revealed incidences of 0.2% (3/1867; 95% CI: 0–0.3%), 2.7% (51/1867; 95% CI: 2.0–3.5%), and 0.1% (1/1867; 95% CI: 0–0.2%), respectively (Table 1). We did not detect any *Anaplasma* spp. or *Borrelia* spp.

**Table 1:** Prevalence of hemoplasma species in commercial pig farms of Korea from 2014 to 2018

Group		No. tested	*Mycoplasma suis*			*Mycoplasma parvum*			Novel hemotropic *Mycoplasma haemosuis*		
			No. positive (%)	95% CI	Chi-square test	No. positive (%)	95% CI	Chi-square test	No. positive (%)	95% CI	Chi-square test
Breed	Breeding pigs	720	2 (0.3)	0–0.7	*χ*_(1)_^2^ = 1.002, *P* = 0.3169	21 (2.9)	1.7–4.2	*χ*_(1)_^2^ = 0.1510, *P* = 0.6976	1 (0.1)	0–0.4	*χ*_(1)_^2^ = 1.594, *P* = 0.2068
	Fattening pigs	1147	1 (0.1)	0–0.3		30 (2.6)	1.7–3.5		0	0	
Region	Northern	591	1 (0.2)	0–0.5	*χ*_(3)_^2^ = 0.8050, *P* = 0.8483	6 (1.0)	0.2–1.8	*χ*_(3)_^2^ = 19.432, *P* = **0.0002**	0	0	*χ*_(3)_^2^ = 1.064, *P* = 0.7859
	Central	306	0	0		3 (1.0)	0–2.1		0	0	
	Southern	905	2 (0.2)	0–0.5		39 (4.3)	3.0–5.6		1 (0.1)	0–0.3	
	Jeju Island	65	0	0		3 (4.6)	0–9.7		0	0	
Total		1867	3 (0.2)	0–0.3		51 (2.7)	2.0–3.5		1 (0.1)	0–0.2	

*Abbreviations*: 95% CI, 95% confidence interval

*Note*: Significant *P*-value (*P* < 0.05) is indicated in bold

No statistically significant differences in *Mycoplasma* prevalence were observed between breeding and fattening pigs; however, breeding pigs were more likely to be carriers than fattening pigs. Overall prevalences were as follows: *M. suis* (0.3%, 2/720; *χ*^2^ = 1.002, *df* = 1, *P* = 0.3169); *M. parvum* (2.9%, 21/720; *χ*^2^ = 0.1510, *df* = 1, *P* = 0.6976); and *M. haemosuis* (0.1%, 1/720; *χ*^2^ = 1.594, *df* = 1, *P* = 0.2068). Geographically, the prevalence of *M. parvum* was significantly increased in the south (*χ*^2^ = 19.432, *df* = 3, *P* = 0.0002), whereas *M. suis* was detected in both northern and southern regions (*χ*^2^ = 0.8050, *df* = 3, *P* = 0.8483). *Mycoplasma haemosuis* was only detected in the southern region (0.1%, 1/905; *χ*^2^ = 1.064, *df* = 3, *P* = 0.7859).

### Molecular and phylogenetic analyses

Nucleotide alignment and phylogenetic analysis were performed using representative samples selected from different years and rearing regions. Sequences of the *16S* rRNA gene from three *M. suis* (14-GN-32, 15-GN-22 and 16-GG-108; accession nos. MK492380, MK492381 and MK492382, respectively), eight *M. parvum* (14-GN-16, 14-GN-624, 15-GN-123, 16-GG-12, 16-CN-16, 18-JJ-43, 18-GB-16 and 18-JJ-19; accession nos. MK492383–MK492390, respectively) and one *M. haemosuis* (14-GN-19; accession no. MK492379) strains were analyzed. Sequence alignment revealed that three *M. suis* and eight *M. parvum* strains from the present study were 98.9–100% and 97.5–100% homologous to one another, respectively.

**Fig. 2:**
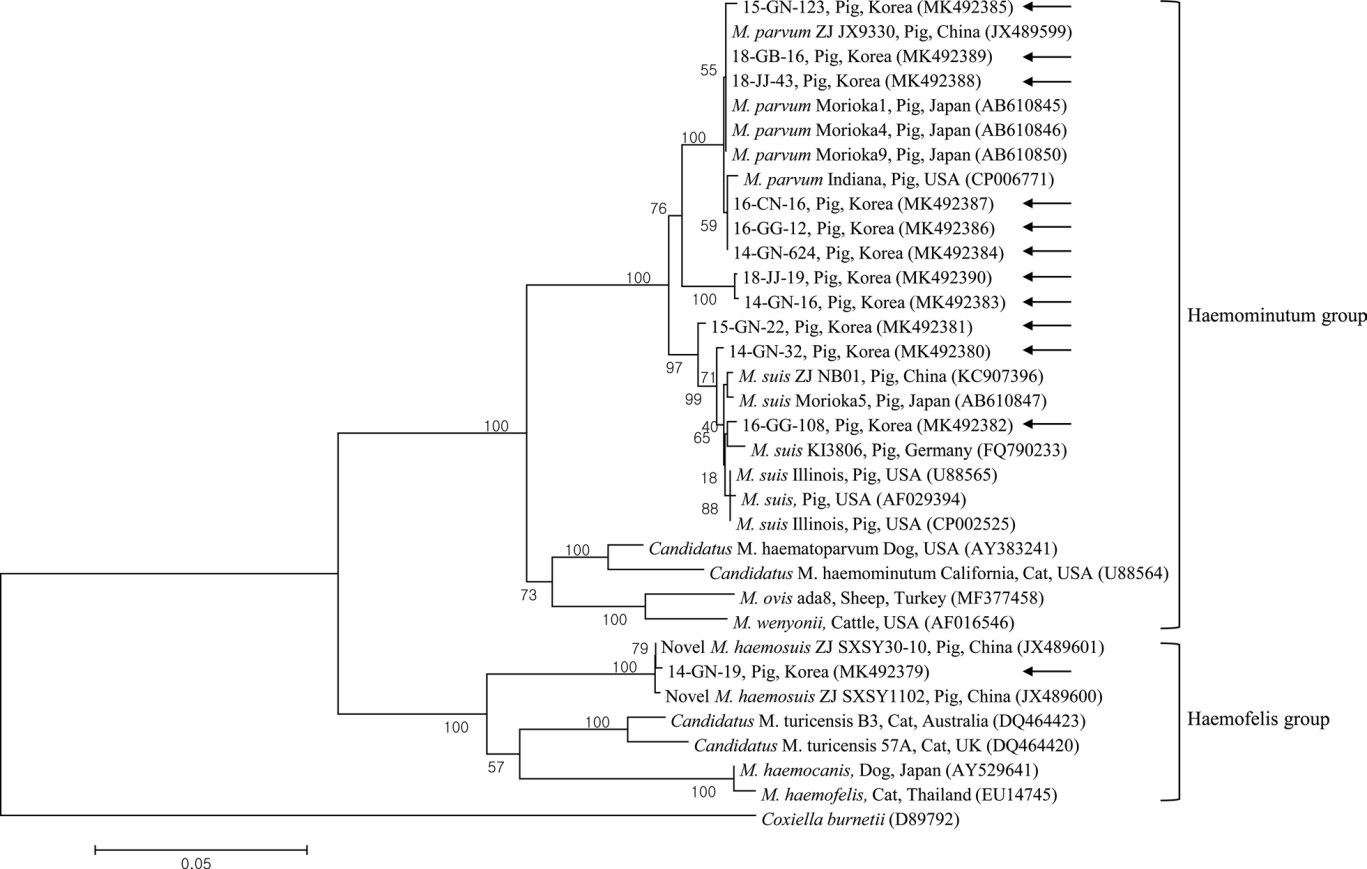
Phylogenetic tree of *Mycoplasma* spp. based on the *16S* rRNA gene. This tree was constructed using the maximum likelihood method. Arrows indicate the sequences generated in the present study. GenBank accession numbers are shown in parentheses. *Coxiella burnetii* (GenBank: D89792) was used as the outgroup. Numbers at branches indicate bootstrap support levels (1000 replicates) and the scale bar indicates the number of substitutions per site

Phylogenetic analysis using the *16S* rRNA (Fig. 2) gene demonstrated that the *Mycoplasma* species detected in this study clustered with those from the GenBank database. Results of nucleotide sequence alignment in the present study also showed high identity with those reported from other countries. The three *M. suis 16S* rRNA sequences shared high identity with those of *M. suis* isolated from pigs in China, Japan, Germany and the USA at 99.6% (GenBank: KC907396), 99.6% (GenBank: AB610847), 99.4% (GenBank: FQ790233) and 99.3% (GenBank: AF029394), respectively. The eight *M. parvum 16S* rRNA sequences shared 100% identity with those of *M. parvum* isolated from pigs in China (GenBank: JX489599) and Japan (GenBank: AB610846), and 99.6% identity with pig isolates from the USA (GenBank: CP006771). The single *M. haemosuis 16S* rRNA sequence shared 93.0–99.9% identity with sequences of *Mycoplasma* spp. isolated from pigs in China (99.8%, GenBank: JX489600; 99.9%, GenBank: JX489601), as well as cats in Australia (93.1%, GenBank: DQ464423) and the UK (93.0%, GenBank: DQ464420).

## Discussion

Natural transmission routes of *M. suis* remain largely unknown [22]. It has been found, however, that the parasite can be shed in saliva, nasal and vaginal secretions and urine from infected animals, and can contaminate dust, water and food, favoring rapid spread of the pathogen [22]. The poor hygiene conditions in domestic pig farms create a favorable environment for the development of arthropods, which might be significant vectors of *M. suis* transmission among animals [14]. Mechanical transmission of *M. suis* by the stable fly *Stomoxys calcitrans* and the yellow fever mosquito *Aedes aegypti* has also been reported in pigs [23]. Other situations favorable to *M. suis* transmission between pigs, including intake of food contaminated with infected blood and reuse of needles by farm workers, may be additional risk factors associated with swine hemoplasmosis in China [14]. Importantly, infection with *M. suis* has also been reported among pig farm workers in China [14], suggesting that this hemoplasma species should be regarded as a potential zoonotic pathogen and a threat to public health.

In this study, three (0.2%) and 51 (2.7%) of the 1867 domestic pigs tested positive for *M. suis* and *M. parvum*, respectively. As *16S* rRNA gene sequences of *M. suis* and *M. parvum* have high sequence identity [24], it is difficult to design PCR primers distinguishing the two species. In a previous study, comparative analysis of *16S* rRNA sequences showed the existence of two different *M. suis* subtypes in wild boars: one (group A) was closely related to the known American and European *M. suis* isolates and the other (group B), which had only 96.9% identity with group A, formed an independent sub-cluster within *M. suis* isolates from China [15]. Based on these data, group B isolates that were morphologically identified as *M. suis* but were closer to *M. parvum* may need to be reclassified as *M. parvum*, and conclusions of previously published studies may need to be reevaluated when appropriate molecular data become available. The sequence of the *M. suis 16S* rRNA gene was first determined in the Illinois strain (GenBank: U88565) in 1997 [25], whereas the complete genome sequence of this strain (GenBank: CP002525) was determined in 2011 [26]. However, *M. parvum* cannot be maintained *in vivo*, which delayed the identification of this species, and *16S* rRNA and RNase P RNA genes in *M. parvum* Morioka 1, 4 and 9 strains were sequenced much later [24]. The complete genome sequence of the *M. parvum* Indiana strain (GenBank: CP006771) was determined in 2014 [11].

Interestingly, in the present study, comparative analysis of *16S* rRNA sequences revealed the existence of *Mycoplasma* species other than *M. suis* or *M. parvum* in only one (0.1%) pig. This novel hemotropic *M. haemosuis*, which was recently detected in pigs from China [12], is not an officially recognized species and is not in the List of Prokaryotic Names with Standing in Nomenclature [6]. Results from our *16S* rRNA-based phylogenetic analysis suggest that this putative novel porcine hemoplasma species is a member of a new cluster genetically related to “*Candidatus* M. turicensis” isolates from the cat, which, together with *M. haemocanis* and *M. haemofelis*, belong to the Haemofelis subgroup, whereas *M. suis* and *M. parvum*, together with *M. ovis*, *M. wenyonii*, “*Candidatus* M. haemominutum” and “*Candidatus* M. haematoparvum”, belong to the Haemominutum subgroup. This division of hemoplasma species into these two subgroups has previously been confirmed by *16S* rRNA sequencing data [12, 27, 28].

Results of this study indicated that breeding pigs are more likely to be infected with *Mycoplasma* than fattening pigs, which is consistent with a previous report that sows presented with higher rates of *M. suis*, *M. parvum* and the novel hemotropic *M. haemosuis* than growing pigs [12]. These observations may reflect the fact that the risk for infection increases with herd age, as the chances of contact with *M. suis*-contaminated sources grow with time [29]. Moreover, *M. suis* may cause immunosuppression in adult pigs, which favors co-infection and secondary infection [29]. Regarding geographical distribution, in this study, *M. suis* was detected in both northern and southern regions, whereas the prevalence of *M. parvum* was significantly higher in the southern region, and the novel hemotropic *M. haemosuis* was exclusively detected in the southern region. These data suggest the prevalence of *Mycoplasma* spp. differs among geographical locations, and is likely associated with the habitat and distribution of their vectors. Of note, the prevalence of *M. suis*-infected pigs was also higher in the southern region of Germany [9].

## Conclusions

To the best of our knowledge, this is the first nationwide, large-scale study on the molecular detection of *Mycoplasma* infection among Korean domestic pigs. Our results indicate that infections with *M. suis*, *M. parvum* and the novel hemotropic *M. haemosuis* are widespread in Korea. Unfortunately, a lack of information concerning *Mycoplasma* spp. carriage by domestic pigs may delay the administration of effective measures to limit the spread of hemoplasma infection. Therefore, continuous monitoring and control strategies should be implemented in pig farming to prevent the spread of hemoplasmas through herds. Additional epidemiological studies are required to identify the reservoirs and vectors of *Mycoplasma* spp., to reduce or halt the spread of these pathogens among domestic pigs.

## Data Availability

Data supporting the conclusions of this article are included within the article. The newly generated sequences were submitted to the GenBank database under the accession numbers MK492379-MK492390. The datasets used and/or analyzed during the present study are available from the corresponding author upon reasonable request.

## Abbreviations

95% CI: 95% confidence interval
df: degrees of freedom
IACUC: Institutional Animal Care and Use Committee
KNU: Kyungpook National University
